# Revision surgery after triple semicircular canal plugging and morphologic changes of vestibular organ

**DOI:** 10.1038/s41598-019-55810-7

**Published:** 2019-12-18

**Authors:** Daogong Zhang, Yafeng Lv, Yuechen Han, Gaoying Sun, Yawei Li, Xiaofei Li, Lixin Sun, Ruozhen Gong, Zhaomin Fan, Haibo Wang

**Affiliations:** 1 Department of Otolaryngology-Head and Neck Surgery, Shandong Provincial ENT Hospital, Shandong University, Jinan, 250022 P.R. China; 2 Department of Radiology, Shandong Provincial ENT Hospital, Shandong University, Jinan, 250022 P.R. China; 3 Shandong Medical Imaging Research Institute, Jinan, 250022 P.R. China

**Keywords:** Diseases, Signs and symptoms

## Abstract

This study aims to investigate the causes of vertigo relapse in patients with Meniere’s disease (MD) who had undergone triple semicircular canal plugging (TSCP) and explore the morphologic changes of vestibular organ through revision surgery. Eleven intractable MD patients who underwent TSCP initially and experienced episodic vertigo recurrence later, were enrolled. All patients accepted revision surgery, including seven cases who underwent labyrinthectomy and four cases who underwent repeat TSCP. Pure tone test, caloric test and video-head impulse test (v-HIT) were used to evaluate audiological and vestibular functions. Specimens of canal plugging materials and vestibular end organs were collected from patients who underwent labyrinthectomy during revision surgery. Mineralization and other histological characteristics of canal plugging materials were evaluated by von Kossa staining. Incomplete occlusion or ossification was observed in the semicircular canals (SCs) of all eleven patients, with all three SCs affected in three, the superior SC in five patients, the horizontal SC in two and the posterior SC in one. The results of v-HIT were in accordance with findings discovered intraoperatively. Few mineralized nodules and multiple cavities were found in the von Kossa-stained canal plugging materials. Incomplete occlusion or ossification of SCs was the principal cause of vertigo recurrence in MD patients who underwent TSCP. v-HIT was helpful in determining the responsible SCs.

## Introduction

Meniere’s disease (MD) is a common chronic inner ear disease, characterized by intermittent episodes of vertigo, fluctuating sensorineural hearing loss, tinnitus and aural pressure. Its prevalence ranges from 3.5 to 513 per 100,000 persons, with the most current estimate being approximately 200 per 100000^1^. Because its definitive pathogenesis remains undetermined, there is currently no cure for this disorder. Initial medical treatment is successful in treating symptoms in about 80% of patients, whereas surgical methods, such as endolymphatic sac surgery, vestibular neurectomy and labyrinthectomy, are considered when medical treatment fails to control the vertigo. Semicircular canal plugging (SCP), first used to treat patients with intractable benign paroxysmal positional vertigo^2^, has been applied to patients with intractable peripheral vertigo recently^3,4^. Lateral canal plugging in 28 patients with MD resulted in vertigo control in 21 (75%)^3^. Triple semicircular canal occlusion in three patients with MD who had undergone unsuccessful endolymphatic sac decompression or mastoid shunt resulted in complete control of vertigo in two patients and substantial control in the third^4^.

We originally performed triple SCP (TSCP) to treat patients with intractable MD, as defined by the 1995 criteria of the American Academy of Otolaryngology-Head and Neck Surgery (AAO-HNS)^5^, finding that this method yielded promising results in controlling vertigo attacks over 2 years^6^. Despite the high rate of SCP effectiveness in the treatment of MD, some patients experience vertigo relapse, for reasons as yet unknown. To date, we have performed TSCP on 527 patients with intractable MD. Of these, 15 patients (2.8%) experienced vertigo relapse and 11 patients (2.1%) underwent reoperation. The clinical data of these eleven MD patients were analyzed to determine the causes of vertigo relapse after TSCP.

## Results

All eleven patients experienced post-TSCP attacks of vertigo at least twice per month, with each attack lasting more than 20 min, suggesting that TSCP was ineffective in controlling their vertigo. The time of recurrence was 7 months to 60 months after TSCP, with average time of 17.5 months. The symptoms of recurrence include vertigo and imbalance. Compared with symptoms before TSCP, the symptoms of recurrence was better in 6 patients, worse in 2 patients and not change in three patients. All eleven patients agreed to undergo reoperation, with seven undergoing labyrinthectomy and four undergoing repeat TSCP. The criteria for choosing labyrinthectomy or TSCP depended on the hearing level of patients. When the pure-tone threshold ≥80 dB, labyrinthectomy was chosen, otherwise, TSCP was used for reoperation. Vertigo was well controlled in all eleven patients after revision surgery, with none experiencing relapse after two-years follow-up.

**Figure 1:**
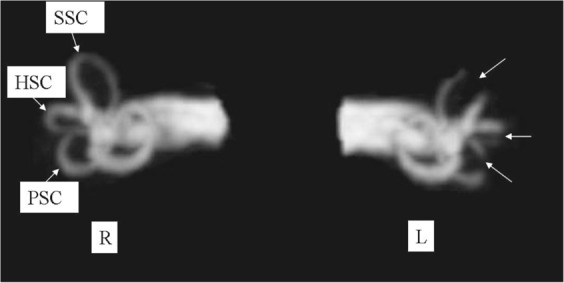
Magnetic resonance hydrography of the labyrinths of a 58-year-old patient with Meniere’s disease in the left ear 2 years after TSCP. Three-dimensional fast imaging employing steady acquisition (3D-Fiesta) imaging showed absence of endolymph fluid in the position of plugging of the three semicircular canals of the affected left ear (arrows), whereas endolymph fluid of the right, healthy ear was normal. Abbreviations: SSC, superior semicircular canal; HSC, horizontal semicircular canal; PSC, posterior semicircular canal; R, right ear; L, left ear.

Before revision surgery, magnetic resonance labyrinthography detected defects in each occluded SC of all eleven patients (Fig. 1), and caloric tests showed hypofunction of each occluded horizontal SC in all eleven patients. v-HIT showed partially abnormal plugged SCs (Table 1). Hearing levels were phase III(41~70 dB nHL) in one patient, and phase IV(>70 dB nHL) in ten.

**Table 1:** General clinical data and findings during revision surgery of eleven patients who failed TSCP.

No.	Sex	Age (years)	Duration of illness (years)	Side of surgery	Incompletely occluded or ossified SCs	v-HIT gain in normal range before revision surgery
1	Female	42	5	Right	SSC	SSC
2	Male	42	9	Left	SSC	SSC
3	Female	71	5	Left	All 3 SCs	PSC
4	Female	59	23	Right	SSC	SSC
5	Male	59	6	Left	HSC	HSC
6	Female	47	7	Left	SSC	SSC
7	Female	57	10	Left	All 3 SCs	SSC
8	Female	53	6	Left	SSC	SSC
9	Male	36	14	Right	HSC	HSC
10	Female	50	10	Left	All 3 SCs	HSC and SSC
11	Female	70	6	Right	PSC	PSC

Abbreviations: SSC, superior semicircular canal; HSC, horizontal semicircular canal; PSC, posterior semicircular canal; SC, semicircular canal.

**Figure 2:**
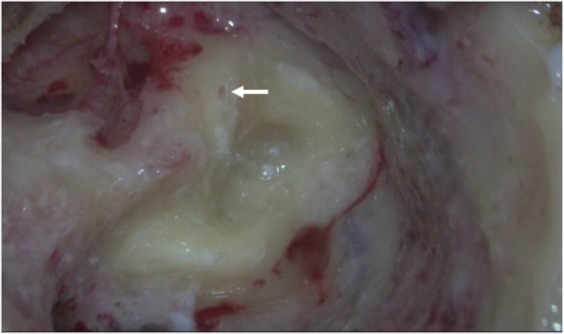
Mastoid cavity during labyrinthectomy of a patient with Meniere’s disease of the left ear who had undergone TSCP two years earlier. The superior semicircular and posterior canals were occluded successfully and the cavity of the canals was completely plugged. The plugging fascia had been fibrotic and ossifying and the lumens of the canals had disappeared. The horizontal semicircular canal was not plugged completely and endolymph in the horizontal semicircular canal was not successfully blocked (as shown by the arrow). Abbreviations: SSC, superior semicircular canal; HSC, horizontal semicircular canal; PSC, posterior semicircular canal.

Reoperation revealed incompletely plugged or ossified SCs in all eleven patients. The superior SC was affected in five patients, the horizontal SC in two, the posterior SC in one and all three SCs in three. Small amounts of residual endolymph fluid were observed in the occlusion areas of incompletely plugged SCs, but these amounts could not be detected by magnetic resonance hydrography of the labyrinth. The temporalis fasciae remained in these canals, but were dissociated, did not adhere to the canal, and were easy to remove (Fig. 2). In the completely plugged SCs, the plugging fasciae were fibrotic and ossifying and the lumens of the canals had disappeared. The results of v-HIT were often normal in patients with incompletely plugged SCs, but were often abnormal in patients with completely plugged SCs. Findings in eight (72.7%) patients were in accordance with the results of v-HIT, whereas findings in the other three patients were in partial accordance with the results of v-HIT (Table 1).

**Figure 3:**
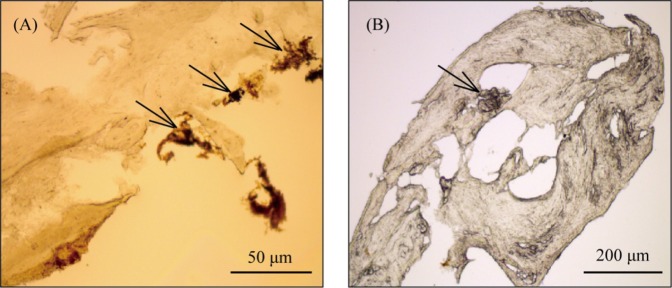
Mineralization of canal plug material (**A**,**B**) obtained from patients who had undergone labyrinthectomy as revision surgery for failed TSCP. Arrows indicate mineralized nodules.

According to this study, von Kossa staining of material from incompletely occluded SCs showed few mineralized nodules or multiple cavities (Fig. 3), indicating that incomplete plugging and/or ossification may have contributed to the recurrence of vertigo after TSCP. These findings were consistent with those of v-HIT.

**Figure 4:**
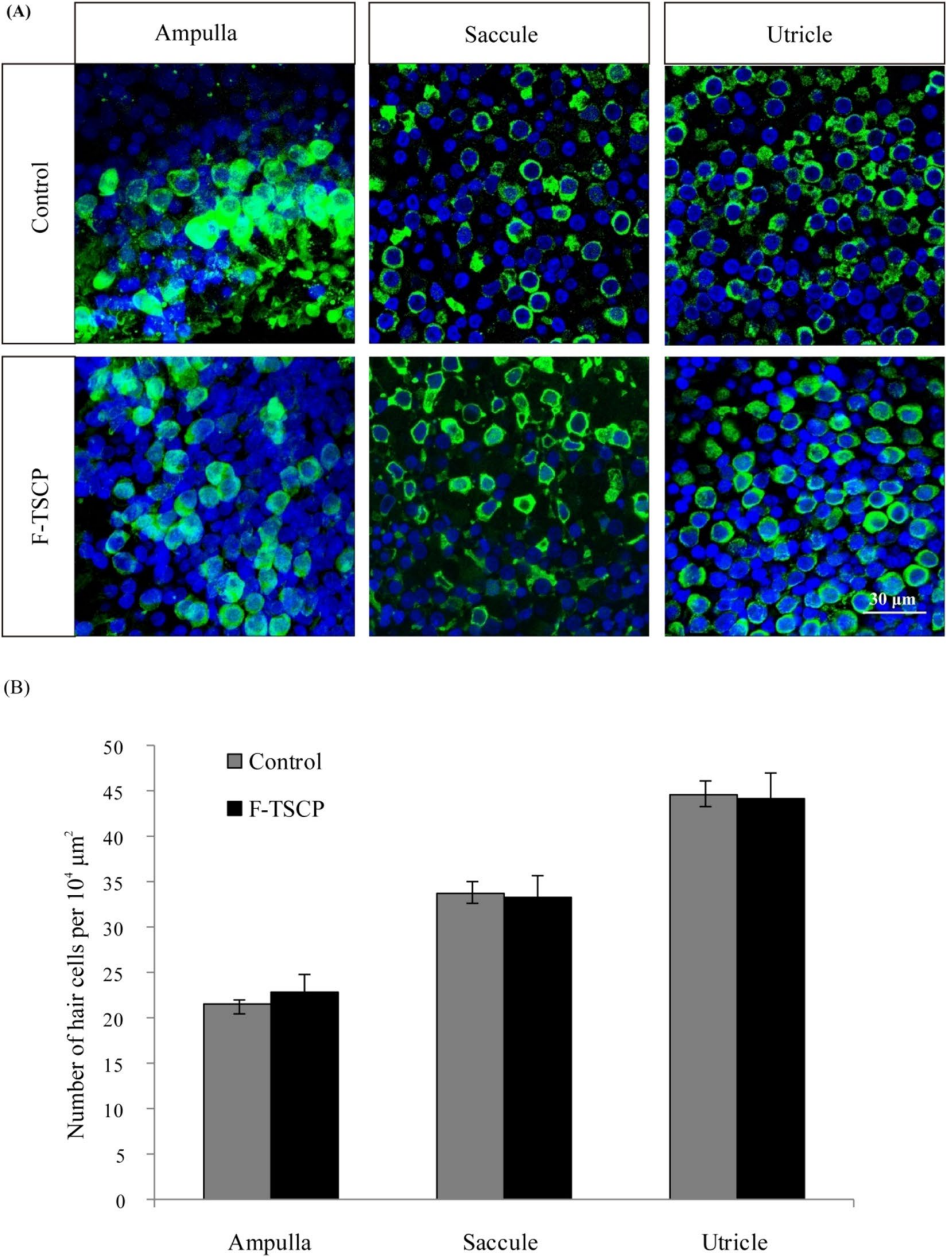
Immunostaining of hair cells from vestibular end organs. (**A**) Samples were stained with antibody to myosin VIIa (green, a marker of hair cells) and DAPI (blue, a marker of nuclei). Control, samples obtained from acoustic neuroma patients. F-TSCP, samples obtained from patients who had undergone labyrinthectomy as revision surgery for failed TSCP. (**B**) Quantification of the average number of hair cells per 10^4^ µm^2^ in vestibular end organs.

Immunofluorescence showed that the number of hair cells in vestibular end organs was similar in the patients who underwent revision surgery and control patients with acoustic neuroma (Fig. 4).

## Discussion

Although there is currently no cure for MD, more than 85% of these patients are helped by either changes in lifestyle or medical treatment^7^. Operative measures are considered when medical treatment has failed to control the vertigo. Surgical methods of choice for intractable patients non-responsive to medication include endolymphatic sac surgery, labyrinthectomy and vestibular neurectomy. Endolymphatic sac surgery is fairly conservative, with a vertigo control rate of 70%^8^. Labyrinthectomy has a higher vertigo control rate, but at the cost of total hearing loss in the affected ear. Vestibular neurectomy is a type of intracranial surgery, with higher risks, lowering the number of patients willing to undergo this procedure^9^. SC plugging is a recently introduced method of treating MD^3,4,6^. TSCP did not affect cochlear function in normal experimental animals or in the guinea pig model of endolymphatic hydrops^10,11^. Two years after TSCP, 78 of 79 (98.7%) patients with MD continued to show effective control of vertigo after 2 years according to our study^6^. To date, the specific mechanism by which SCP controls vertigo attacks remains undetermined. In this study, caloric test showed dysfunction of occluded SCs in all 11 subjects by the end of follow-up, indicating that TSCP controlled vertigo attacks by reducing the function of SCs. Plugging of an SC may block endolymph fluid in this canal, as well as greatly reduce the movement of endolymph within an SC. This may lead to minimal stimulation of hair cells during angular motion and changes in endolymph pressure. In our study, the operated ears failed to respond to caloric stimulation, suggesting that fluid movement induced by caloric stimulation was blocked by TSCP. Despite the high rate of SCP effectiveness in the treatment of MD, some patients experience vertigo relapse, for reasons as yet unknown.

The present study showed that SCP blocked the flow of endolymphatic fluid during reoperation, which may explain the mechanisms underlying the effectiveness of SCP in the treatment of MD. We also found that all nine patients had one or more incompletely occluded or ossified SCs. von Kossa staining of canal material showed incomplete occlusion and/or ossification, further validating the results of v-HIT. In addition to explaining the reason for vertigo recurrence, these findings indicated that complete occlusion was essential for ensuring the effectiveness of SCP. To prevent incomplete occlusion or ossification, we think several suggestions might be helpful. Firstly, the size of the fenestration should be appropriate. According to our experience, 2 mm in diameter of the fenestration is suitable, while too large of the fenestration might cause incomplete plugging. Secondly, the plugging material should be changed. We found that the combination of temporalis fasciae with bone dust was better than the temporalis fasciae only. Recurrence may be due to the transfer of pressure to the ampulla through the small amount of residual endolymph fluid, which was not completely blocked in the occluded canal. Additionally, these patients experienced severe vertigo after labyrinthectomy, further indicating that TSCP had preserved the function of the operative ampulla, but that this function was destroyed during labyrinthectomy. Immunofluorescence showed that numbers of ampulla hair cells were similar in the patients who underwent revision surgery and in the control group of patients with acoustic neuroma, suggesting that TSCP did not affect the morphology of the ampulla. In addition, the numbers of saccule and utricle hair cells were similar in the two groups, indicating that TSCP did not affect otolith function.

Chemical labyrinthectomy had been widely used to treat intractable MD in recent years^12,13^. Compared with TSCP, the principle of chemical labyrinthectomy was to destroy the vestibular function to control vertigo. So the function of vestibular hair cells was impaired in chemical labyrinthectomy. However, TSCP had no damage to hair cells of vestibular end organs. It was just through blocking the movement of endolymph within semicircular canals to control vertigo. So, TSCP was less destructive compared with chemical labyrinthectomy theoretically. Compared with labyrinthectomy or vestibular neurectomy, vestibular compensation accomplished more rapidly in TSCP due to the preservation of the function of otolith organ and ampulla hair cells. Similarly, recovery was found to be more rapid in cats that underwent TSCP than in those that underwent labyrinthectomy^14^. Additionally, the effect of vertigo control was as good as labyrinthectomy or vestibular neurectomy in TSCP according to our study^6^.

The presence of residual endolymph fluid in the plugging area of incompletely occluded SCs may have been due to absorption of the temporalis fascia and reopening of the lumen. Moreover, we found that the findings in eight patients were in accordance with the results of v-HIT before reoperation. That is, completely occluded SCs usually showed abnormal results on v-HIT, whereas incompletely occluded SCs usually showed normal results on v-HIT. This provides a feasible method for determining the SCs responsible for vertigo relapse. Incompletely occluded SSCs were observed in eight of the eleven patients, a finding that may be related to the anatomic characteristics of the SCs. HSCs and PSCs were occluded at right angles, whereas SSCs was occluded at acute angles, making complete blocking of SSCs more difficult. The limited ability of magnetic resonance labyrinthography to determine responsible SCs may be due to the limited resolution of MRI. This method may be able to distinguish occlusion from non-occlusion but was unable to distinguish incomplete from complete occlusion or detect the small amount of endolymph fluid in the canals of patients with recurrence of vertigo.

In conclusion, TSCP showed definite long-term effects in the treatment of patients with intractable MD. TSCP had no damage to hair cells of vestibular end organs. Vertigo relapse, observed in a few patients after surgery, may have been due to the incomplete occlusion of SCs. Complete occlusion or ossification of SCs is essential for the effectiveness of TSCP. According to our study, v-HIT may be helpful in determining the responsible SCs, whereas magnetic resonance labyrinthography had limited value.

## Materials and Methods

### Patients

Eleven patients with MD (three men, eight women; age range 36–71 years; mean age 53.3 years), evaluated at the vertigo clinic of Shandong Provincial ENT Hospital affiliated to Shandong University from Dec. 2010 to Jun. 2017, were retrospectively analyzed. All eleven patients had been clinically diagnosed with definite MD, as defined by the 1995 criteria of the American Academy of Otolaryngology-Head and Neck Surgery (AAO-HNS)^5^, underwent TSCP, and were followed up for at least 2 years. All eleven patients experienced post-TSCP attacks of vertigo and accepted revision surgery.

Before revision surgery, the audiologic and vestibular functions were assessed by pure tone tests, caloric tests, cervical vestibular evoked myogenic potential (cVEMP) and video-head impulse tests (v-HIT), and membranous labyrinth morphology was evaluated by magnetic resonance labyrinthography. Clinical samples including canal plugging material and vestibular end organs (ampulla, saccule and utricle) were obtained from patients who underwent labyrinthectomy as the revision surgery. Canal plugging material was histologically characterized by von Kossa staining, and the morphology of hair cells in vestibular end organs was evaluated by immunofluorescence. Three patients (one men, two women; age range 40–65 years; mean age 54.3 years) with acoustic neuroma were selected as control group. The study was approved by the Ethics Committee of Shandong Provincial ENT Hospital affiliated to Shandong University and all patients provided written informed consent. Our study involving humans is in compliance with the Declaration of Helsinki.

## Main Outcome Measures

### Evaluation of hearing

Hearing function was evaluated by pure tone audiometry, based on the four-tone average of hearing levels at 0.5 kHz, 1 kHz, 2 kHz and 3 kHz, according to 1995 AAO-HNS criteria^5^.

### Caloric test

Bithermal caloric tests were performed as described before^6^. Briefly, each ear was irrigated alternatively with a constant flow of air at 24 °C and 49 °C for 40 seconds. The response was recorded over 3 minutes, with a 7-minute interval between stimuli to avoid cumulative effects. A video-based system was used (Ulmer VNG, v. 1.4; SYNAPSYS, Marseille, France) to acquire and analyze eye responses. The maximum slow-phase velocity (SPV) of nystagmus after each irrigation was calculated, and unilateral weakness (UW) was determined according to Jongkees’ formula. In our laboratory, a UW less than 20% was considered normal^6^.

### v-HIT

Head impulse tests were performed using a v-HIT device (Ulmer II Evolution, France). Subjects were seated 1.5 m in front of a wall on which three small red-colored targets (one right ahead, the other two deviating 20° from the central line) were affixed at eye level. Subjects were instructed to fixate on a target and more than three head impulses (amplitude 15°~20°, duration 100~200 ms) were performed for each direction. Head and eye movement were recorded using a video-oculography system and the VOR gain was automatically calculated. Angular acceleration thresholds were set at 2000°/s^2^ for horizontal tests and 2000°/s^2^ for vertical tests. VOR gains under 0.8 for horizontal tests and under 0.7 for vertical tests were considered abnormal.

### Magnetic resonance hydrography of labyrinth

Magnetic resonance imaging (MRI) scans were acquired using a 1.5 Tesla MR unit (GE-signal, USA) and a circular temporal coil as described before^6^. Briefly, three-dimensional fast imaging employing steady acquisition (3D-Fiesta) imaging was performed and the scan parameters for the 3D-Fiesta included a repetition time of 1.7 ms, an effective echo time of 5.0 ms, a matrix size of 320 × 384, and with 30 axial 0.8-mm thick slices to cover the labyrinth and internal auditory canal with a 180-mm square field of view^6^. The number of excitations was two, and the scan time was 4 minutes^6^. All MRI raw data were sent to a GE post-processing workstation and the maximum intensity projection (MIP) was used to reconstruct the structure of the labyrinth. MIP- reconstructed images were rotated once from 0 degrees in 15 degree steps to obtain multi-azimuth and multi-view images of the labyrinth and internal auditory canal^6^.

### von Kossa staining

Canal plugging material samples were obtained from the SCs of patients who had undergone labyrinthectomy. The samples were washed twice with ice-cold PBS, fixed in 4% paraformaldehyde for 2 h at room temperature, and subjected to gradient dehydration with 15%, 20%, and 30% sucrose in PBS. The dehydrated samples were embedded in Tissue-tek O.C.T. compound (Sakura, Japan) and cryosectioned to yield samples 7 µm thick. Calcium deposits were assessed histochemically by von Kossa staining (Genmed, USA), according to the supplier’s protocols. The samples were positioned under UV light for 1 h in the dark, rinsed 3 times with double-distilled water and treated with 5% sodium thiosulfate to remove background staining. After three washes with PBS, the samples were air-dried and examined under the microscope.

### Immunohistochemistry

Vestibular end organs, including ampulla, saccule and utricle, were obtained from patients who had undergone labyrinthectomy. These samples were washed twice with ice-cold PBS, fixed in 4% paraformaldehyde and permeabilized with 1% TritonX-100 (Sigma, USA) in PBS. After one-hour blocking in PBT-1 [0.1% TritonX-100, 8% donkey serum, 1% bovine serum albumin (BSA), and 0.02% sodium azide in PBS] at room temperature, the samples were incubated overnight at 4 °C with rabbit anti-myosin VIIa primary antibody (Proteus, USA) diluted in PBT-1. The samples were washed and incubated at room temperature for 1 h with FITC-conjugated secondary antibody (Invitrogen, USA), along with DAPI (Sigma), in PBS supplemented with 0.1% Triton X-100 and 1% BSA. The samples were subsequently mounted and viewed under a laser scanning confocal microscope (Leica, Germany).
